# Cardiorespiratory coupling is associated with exercise capacity in patients with chronic obstructive pulmonary disease

**DOI:** 10.1186/s12890-021-01400-1

**Published:** 2021-01-12

**Authors:** Yu-Chen Huang, Ting-Yu Lin, Hau-Tieng Wu, Po-Jui Chang, Chun-Yu Lo, Tsai-Yu Wang, Chih-Hsi Scott Kuo, Shu-Min Lin, Fu-Tsai Chung, Horng-Chyuan Lin, Meng-Heng Hsieh, Yu-Lun Lo

**Affiliations:** 1 Department of Thoracic Medicine, Chang Gung Memorial Hospital, 199 Tun-Hwa N. Rd., Taipei, Taiwan; 2 College of Medicine, Chang Gung University, Taoyuan, Taiwan; 3 Department of Mathematics, Duke University, Durham, NC USA; 4 Department of Statistical Sciences, Duke University, Durham, NC USA

**Keywords:** Heart–lung interaction, Synchrogram index, Six-minute walking distance, Distance saturation product

## Abstract

**Background:**

The interaction between the pulmonary function and cardiovascular mechanics is a crucial issue, particularly when treating patients with chronic obstructive pulmonary disease (COPD). Synchrogram index is a new parameter that can quantify this interaction and has the potential to apply in COPD patients. Our objective in this study was to characterize cardiorespiratory interactions in terms of cardiorespiratory coupling (CRC) using the synchrogram index of the heart rate and respiratory flow signals in patients with chronic obstructive pulmonary disease.

**Methods:**

This is a cross-sectional and preliminary data from a prospective study, which examines 55 COPD patients. K-means clustering analysis was applied to cluster COPD patients based on the synchrogram index. Linear regression and multivariable regression analysis were used to determine the correlation between the synchrogram index and the exercise capacity assessed by a six-minute walking test (6MWT).

**Results:**

The 55 COPD patients were separated into a synchronized group (median 0.89 (0.64–0.97), n = 43) and a desynchronized group (median 0.23 (0.02–0.51), n = 12) based on K-means clustering analysis. Synchrogram index was correlated significantly with six minutes walking distance (r = 0.42, p = 0.001) and distance saturation product (r = 0.41, p = 0.001) assessed by 6MWT, and still was an independent variable by multivariable regression analysis.

**Conclusion:**

This is the first result studying the heart–lung interaction in terms of cardiorespiratory coupling in COPD patients by the synchrogram index, and COPD patients are clustered into synchronized and desynchronized groups. Cardiorespiratory coupling is associated with exercise capacity in patients with COPD.

## Background

In patients with chronic obstructive pulmonary disease (COPD), cardiovascular disease is a prevalent comorbidity and leading cause of death. The prevalence of cardiovascular disease within this population can be attributed to shared risk factors (e.g., cigarette smoking and exposure to noxious gases) as well as oxidative stress and reduced physical activity related to COPD [1–4]. Deleterious pulmonary function (e.g., dynamic hyperinflation and hypoxia) can also impair cardiovascular mechanics in patients with COPD [5–7]. A more comprehensive understanding of the heart–lung interaction could be highly beneficial in efforts to treat patients with COPD [8–10].

Heart–lung interactions can be classified according to the underlying related but different mechanisms: (1) respiratory sinus arrhythmia, (2) cardioventilatory coupling, and (3) respiratory stroke volume synchronization [11]. For example, during inspiration, central inspiratory drive [12] and negative intrathoracic pressure [13] both contribute to an increase in heart rate [14]. Negative intrathoracic pressure promotes filling of the right ventricle and impedes filling of the left ventricle [15]. A decrease in arterial blood pressure tends to increase respiratory rate and tidal volume through the baroreflex [16].

Cardiorespiratory coupling (CRC) is an intuitive method to depict and quantify the complicated heart–lung interaction by calculating the phases ratio between heartbeat and respiration. CRC in healthy subjects has been extensively studied in terms of age-related evolution [17] and its association with sleep stage transitions [18]. CRC grows in strength in the first 180 day after birth [17], and continues to evolve with age. Association of ages and CRC synchronization is different during sleep and rest periods. In healthy adults, CRC synchronization during rest periods is not correlated with age [19, 20]; however, the strength of CRC has been shown to decrease in elderly adults during sleep [21]. CRC has also been linked to obstructive sleep apnea (OSA) [22] and it has been suggested as a tool by which to assess OSA severity. Under the clinical observation of intimate heart–lung interaction in patients with COPD [23], we hypothesized that CRC could provide clinically relevant information. The aim of this study was to apply the synchrogram index to evaluate CRC in patients with COPD, and to cluster patients based on their synchrogram indices.

## Methods

### Study design and patients

**Fig. 1:**
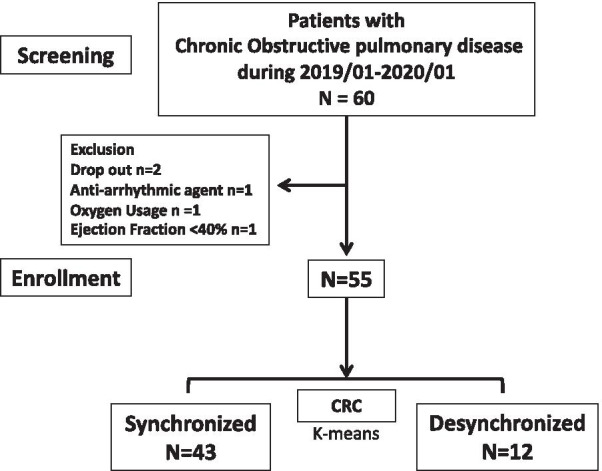
Flow chart

This observational cross-sectional study was based on data obtained from a preliminary prospective study, conducted at Chang Gung Memorial Hospital (CGMH) in Linkou, Taiwan. The sample included patients who underwent regular follow-up as out-patients at CGMH between January 2019 and January 2020. Inclusion criteria included clinical diagnosis of COPD based on the Global Initiative for Obstructive Lung Disease Criteria (GOLD) [24] and post-bronchodilator FEV1 < 80% of the predicted normal value via diagnostic spirometry. Other inclusion criteria were age ≥ 40 years without known heart disease. Exclusion criteria included patients with HFlowEF [heart failure with low ejection fraction (< 40%)], known malignancy, or atrial fibrillation as well as those using oxygen or anti-arrhythmic agents for arrhythmia. All COPD patients underwent cardiac echo analysis, biochemical analysis [eosinophils, high sensitivity C-reactive protein (HS-CRP), and IgE], pulmonary function tests, chest high-resolution computed tomography (HRCT) scanning, a six-minute walking test (6MWT) and a coupling test during the first visit of enrollment. Emphysema was defined based on chest HRCT report from the radiologist and one pulmonologist [25]. Clinical profiles, a list of inhalation medicines, anti-psychotic agents, result of emphysema based on HRCT and acute exacerbation history [26] were also recorded. Although there is one patient who presented high ratio of FEV_1_/FVC before exercise (0.72) and after exercise (0.73), he was not excluded as the spirometry fulfilled the GOLD guideline when he was diagnosed COPD. The remaining 55 patients with COPD [69 (51–84) years old, 54 male] (Fig. 1). All participants signed informed consent prior to enrollment. The study was approved by the Ethics Committee of CGMH (201702150B0).

### Six-minute walking test

The 6MWT was carried out on a straight aisle with smooth surface and of 30 m long. Before the exam, the patients rested in a sitting position, during which spirometry was performed to assess pulmonary function, including the flow volume and tidal volume, based on established guidelines [27]. Oxygen saturation, heart rate, arterial blood pressure, and Borg scale values were recorded to assess the degree of dyspnea. Following this preliminary assessment, the patients were instructed to walk as far as possible within a period lasting six minutes. The patients were permitted to stop and rest when they felt tired or dyspneic, and then encouraged to start again as soon as possible. The instructors avoided walking with the subjects, but rather stood within a designated area to provide encouragement with an even tone at intervals of one minute and at fifteen seconds before the end of the exam, in accordance with to the American Thoracic Society (ATS) guideline [28, 29]. Oxygen saturation and heart rate were recorded in real time while walking. At the end of the exam, walking distance, oxygen saturation, distance saturation product (i.e., the product of nadir saturation during exercise and walking), heart rate and Borg scale were recorded, and patients performed spirometry again after exercise.

### Phase synchronization analysis

#### Instrumentation

Experiments were performed in a quiet room with the temperature maintained at 22–24 °C with all necessary equipment prepared beforehand, including ECG leads, pulse oximeter, a breathing tube (a disposable mouthpiece connecting with end-tidal CO_2_ sensor and flow sensor), and three ACTiwave devices (CamNtech Ltd, Cambridge, UK) connected to a computer running LabChart8 software. Participants were instructed to avoid inhaling short acting bronchodilators for 4 h, taking oral medicines such as beta-2 agonists, xanthene derivatives for 12 h, and consuming alcohol or caffeine-contained drink for at least eight hours prior to the test. Otherwise, participants could intake other foods before the exam. The chest skin was abraded using gel and then cleaned using alcohol to reduce electrode impedance prior to the attachment of electrocardiogram (ECG) electrodes. Prior to the examination, blood pressure, heart rate, and oxygen saturation were recorded. The subjects wore a pulse oximeter on the index finger and ECG electrodes on the chest wall. A breathing tube was inserted into the subject’s mouth with his/her lips sealed around the mouthpiece and a nose clip over the nostrils [30]. Prior to the exam, the subjects were instructed to practice breathing at tidal volume for 1 min and then proceed with the exam when they felt ready. ECG and flow signals were recorded continuously for 5 min using three ACTiwave devices (CamNtech Ltd, Cambridge, UK). The recorded signals were transferred in the European Data Format to LabChart 8 software (ADInstruments, Dunedin, New Zealand), and then exported to text files for analysis.

#### Signal processing and Synchrogram index

**Fig. 2:**
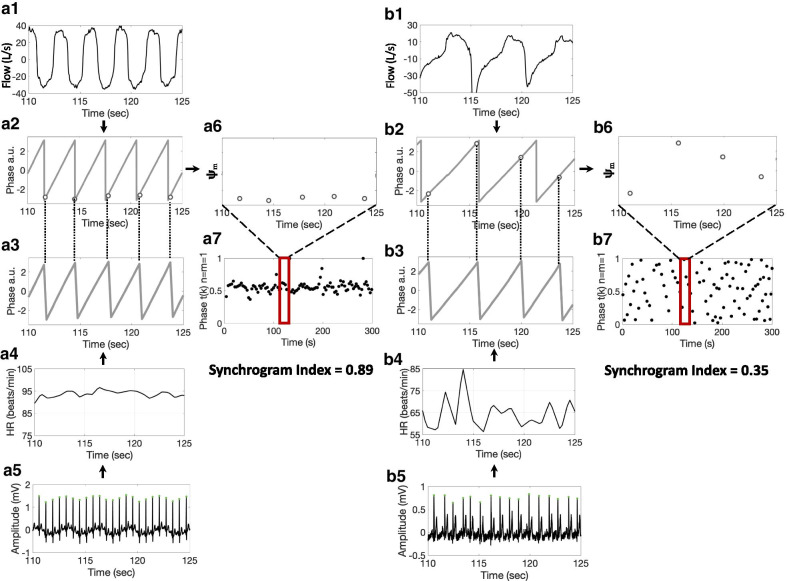
**a1** respiratory flow signal, **a2** phase of the flow signal extracted by the SST, **a3** phase of the IHR extracted by the synchrosqueezing transform (SST), **a4** instantaneous heart rate (IHR), **a5** electrocardiogram, **a6** synchrogram during 110–125 s, **a7** synchrogram and the resulting synchrogram index (0.89) in a synchronized patient. **b1** respiratory flow signal, **b2** phase of the flow signal extracted by the SST, **b3** phase of the IHR extracted by the SST, **b4** IHR, **b5** Electrocardiogram, **b6** synchrogram during 110–125 s, **b7** synchrogram and its synchrogram index (0.35) in a desynchronized patient

R peaks were detected using a standard R peak detection algorithm from the ECG signal (Fig. 2a5, b5). The time differences between consecutive R peaks were calculated, and then converted into an instantaneous heart rate (IHR) time series using the standard interpolation algorithm (Fig. 2a4, b4) [31]. The phase of the respiratory signal (denoted as) was extracted using the synchrosqueezing transform (SST) (Fig. 2a2, b2) [32]. The phase of IHR (denoted as) was extracted by the same method (Fig. 2a3, b3). After obtaining the phases of the IHR and the respiratory signal, the synchrogram was used to quantify the cardiorespiratory coupling [33, 34]. The output is the synchrogram index, which is a non-unit quantity between 0 and 1. When the cardiorespiratory coupling is strong, the synchrogram index is close to 1; Otherwise, it is close to 0.

The synchrogram is a signal processing tool used to depict coupling between two oscillatory signals. In the current study, we first obtained the timestamps [Fig. 2 (dashed line between a2 and a3, b2 and b3)], where the IHR phase attained modulo. We then measured the respiratory phase at as follows: that is, we evaluate the phase of the respiratory signal at [Fig. 2 (circle points at a2 and b2)] Finally, plot against. When the cardiorespiratory coupling is strong, the phase of the respiratory signal at would be fixed for all, and hence we obtain a horizontal stripe in the plot (Fig. 2a6); Otherwise, we obtained scattered points in the plot (Fig. 2b6). The *synchrogram index* [34] is aiming to quantify if the plot is scattered or fixed along a horizontal line. It is defined by, where M is the number of detected cycles in the IHR.

### Statistical analysis

All results are presented as median (range) or mean ± standard deviation. The nonparametric exact two-tailed Mann–Whitney U test was used to determine the statistical significance between two groups of continuous variables, and Fisher’s exact tests were used for categorical variables. Pearson’s correlation coefficient was used to examine the association between 6MWD, distance saturation product (DSP), and other clinical parameters, including the synchrogram index. To avoid variable selection caused by spurious correlations, multivariable regression analysis was based on variables that presented a significant linear relationship (defined as p ≤ 0.05) with 6MWD and DSP. Multivariable regression analysis was performed using the lm function of the R statistical software package. K-means clustering was applied to cluster COPD patients based on their synchrogram indices. Silhouette analysis was performed to select optimal cluster numbers. All reported P values were two-sided, with P < 0.05 considered statically significant. Signals were analyzed using MATLAB. All data were analyzed using R version 3.5.2 (R foundation for statistical computing).

## Results

### Demographic characteristics of patient

Among 55 COPD patients, 54 (98.2%) were male, 49 (89.1%) had smoking history, 36 (65.5%) were in allergic status, 33 (60%) were confirmed with emphysema from chest HRCT, and only 1 (1.8%) patient fulfilled the criteria of Asthma-COPD overlap (ACO) [35, 36]. The median synchrogram indices in the COPD group was 0.87 and the distribution was skewed (range: 0.02–0.97). The median BMI was 24.7 (range 16.7–32.1), the modified medical research council (mMRC) was 1 (range 0–4), and the COPD assessment test (CAT) was 10 (range 2–29). The median ejection fraction was 65.5% (range 52–90), suggesting that there was no patient with heart failure with mid-range ejection fraction (HFmrEF) in this study. However, there were 24 patients (44.3%) presented diastolic dysfunction. The median left atrial size was 34 mm (range 23–46) and E/e′ ratio (the ratio of the trans mitral early peak velocity over early diastolic mitral annulus velocity) was 8.9 (4.5–20.0). In addition, the median eosinophil count was 129 (range 0–615.6), IgE level was 59.7 (2–1652) and 19 (34.5%) patients had a history of acute exacerbation one year prior to enrollment in the study. Most patients used combination therapy of long-acting _2_ agonist (LABA) with long-acting muscarinic antagonist (LAMA) (20 (36.4%)) and triple therapy of LABA with LAMA and ICS (26 (47.3%)) (Table 1).

**Table 1:** Clinical characteristics of COPD patients, clustered into synchronized and desynchronized group

	Total (n = 55)	Synchronized (n = 43)	Desynchronized (n = 12)	p-value
Age, years	69 (51–84)	69 (51–84)	77 (52–84)	0.02
Male, n (%)	54 (98.2)	42 (97.7)	12 (100)	1
Smoker, n (%)	49 (89.1)	38 (88.4)	11 (91.7)	1
Current, n (%)	27 (49.1)	20 (46.5)	7 (58.3)	1
Ex-smoker, n (%)	22 (40)	17 (39.5)	5 (41.7)	1
BMI, kg/m^2^	24.7 (16.7–32.1)	24.2 (16.7–32.0)	26.2 (20.3–30.8)	0.03
Eosinophil Count, number/l	129 (0–615.6)	134.5 (0–615.6)	115.0 (0–329.8)	0.74
Allergic status, n (%)	36 (65.5)	28 (65.1)	8 (66.7)	1
a-IgE, pg/ml	59.7 (2–1652)	65.2 (3.59–1652)	47.0 (2–691)	0.59
HS-CRP, mg/dl	1.7 (0.2–189.7)	1.64 (0.2–37.9)	1.75 (0.2–189.7)	0.65
CAT	10 (2–29)	10 (2–29)	6 (3–29)	0.99
mMRC	1 (0–4)	1 (0–3)	1 (0–4)	0.73
Presence of emphysema, n (%)	33 (60)	27 (62.7)	6 (50)	1
AE history, n (%)	19 (34.5)	16 (37.2)	3 (25)	0.49
Underlying disease				
ACO, n (%)	1 (1.8)	0 (0)	1 (8.3)	0.21
Hypertension, n (%)	17 (30.9)	11 (25.6)	6 (46.2)	0.16
DM, n (%)	8 (14.5)	5 (11.6)	3 (23.1)	0.35
CAD, n (%)	3 (5.5)	3 (7.0)	0 (0)	1
Liver Disease, n (%)	6 (10.9)	6 (14.0)	0 (0)	0.32
Kidney Disease, n (%)	1 (1.8)	0 (0)	1 (7.7)	0.21
Cardiac echo				
Diastolic dysfunction, n (%)	24 (43.6)	20 (46.5)	4 (33.3)	0.51
E/e' ratio	8.9 (4.5–20.0)	8.8 (4.5–13.0)	11.4 (5.2–20.0)	0.06
EF, %	65.5 (52–90)	66.5 (52–90)	64.5 (52–78)	0.33
LA, mm	34 (23–46)	34 (23–46)	33.5 (28–41)	0.99
Drugs				
LABA alone, n (%)	4 (7.3)	3 (7.0)	1 (8.3)	1
LAMA alone, n (%)	1 (1.8)	1 (2.3)	0 (0)	1
LABA + LAMA, n (%)	20 (36.4)	15 (34.9)	5 (41.7)	0.74
LABA + ICS, n (%)	3 (5.5)	2 (4.7)	1 (8.3)	0.54
Triple, n (%)	26 (47.3)	22 (51.2)	4 (33.3)	0.35
OCS, n (%)	5 (9.1)	4 (9.3)	1 (8.3)	1
Anti-psychotic agents, n (%)	2 (3.6)	0 (0)	2 (4.8)	1

Data was presented as mean ± SD or median (range) or number (%). Presence of emphysema was defined as presence of emphysema from high resolution computed tomography. Significantly difference between patients in the synchronized group and desynchronized group was defined as P < 0.05

*COPD* chronic obstructive pulmonary disease, *BMI* body mass index, *HS-CRP* high sensitivity C reactive protein, *CAT* chronic obstructive pulmonary disease assessment test, *mMRC* modified medical research council, *ACO* asthma and COPD overlap, *DM* diabetes mellitus, *E/e′ ratio* the ratio of the transmitral early peak velocity over early diastolic mitral annulus velocity, *EF* ejection fraction, *LA* left atrial, *LABA* Long acting beta agonists, *LAMA* long acting antimuscarinic agents, *ICS* inhaled corticosteroids, *Triple* LABA + LAMA + ICS, *OCS* oral corticosteroids, *AE* acute exacerbation

### ECG, flow signal, CRC data, and synchrogram index

**Fig. 3:**
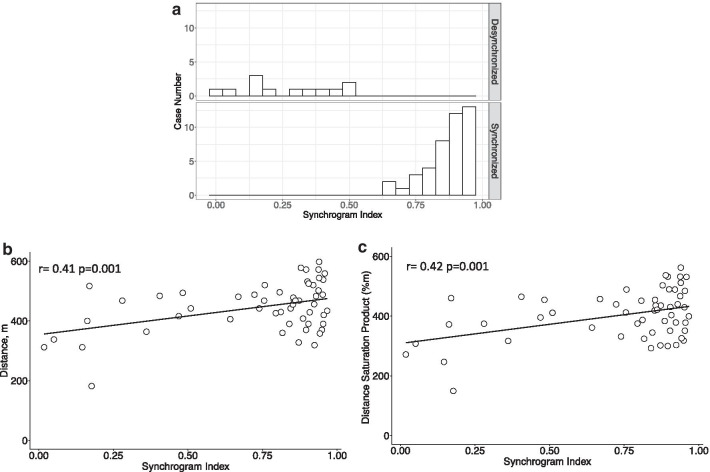
**a** Distribution of synchrogram index in synchronized group and desynchronized group. **b** Scatterplot of synchrogram index against distance (m) from six-minute walking test of all patients. **c** Scatterplot of synchrogram index against distance saturation product (m%) from six-minute walking test of all patient

Figures 2a and 2b illustrate CRC analysis based on the synchrogram of IHR and respiratory flow signals. Since there are no definitions of good or poor synchronization, we applied K-means [37]. Two clusters were identified, i.e., synchronized group (n = 43) and desynchronized group (n = 12) according to the optimal cluster number based on the silhouette analysis. The median synchrogram index values in these two groups were as follows: synchronized group (0.89; 0.64–0.97) and desynchronized group (0.23; 0.02–0.51) (Fig. 3b). Overall, subjects in the synchronized group were younger (69 (51–84) vs 77 (52–84), p = 0.02) and had a lower BMI (24.2 (16.7–32) vs 26.2 (20.3–30.8), p = 0.03). No significance between-group differences were observed in terms of gender, smoking status, allergic status, therapies, or history of acute exacerbation (Table 1).

### Comparing coupling tests with six-minute walking test

In the 6MWT, patients in the synchronized group achieved longer walking distances than did their counterparts in the desynchronized group (468 (328–624) vs. 408 (182–517), unit = m, p = 0.009) and a higher distance saturation product (DSP) (421.2 (255.6–536.6), vs 373.2 (149.2–464.6), unit = m%, p = 0.02) (Table 2). Table 3 lists the correlation between distance and DSP based on clinical parameters recorded during the 6MWT. Age, synchrogram index, CAT, mMRC, and emphysema were all correlated with 6MWD and DSP. The synchrogram index was significantly correlated with distance (r = 0.42, p = 0.001) (Fig. 3c) and DSP (r = 0.41, p = 0.001) (Fig. 3d). In the multivariable regression model, age, mMRC, and synchrogram index were independent variables predictive of distance. Age, synchrogram index, mMRC, emphysema were independent variables predictive of DSP (Tables 4, 5). Distance and DSP could be explained using the following multivariable regression model with the following independent variables: (1) Distance = 671.3 + 93.3Synchrogram Index-3.1Age-37.9mMRC (r^2^ = 0.56, p < 0.0001) (2) DSP = 619.2 + 89.1Synchrogram Index-2.8Age-50.7mMRC-41.1Emphysema (r^2^ = 0.63, p < 0.0001).

**Table 2:** Results of 6MWT in COPD patients, synchronized and desynchronized group

Pre- and post-exercise (6MWT)	COPD (n = 55)	Synchronized (n = 43)	Desynchronized (n = 12)	p-value
Pre-FVC, L	2.6 (1.2–4.4)	2.7 (1.2–4.4)	2.45 (1.3–3.3)	0.28
Pre-FVC, %	78.7 (38–129)	82.5 (39–129)	77 (38–96)	0.89
Pre-FEV_1_, L	1.5 (0.5–2.7)	1.39 (0.6–2.7)	1.43 (0.5–2.0)	0.49
Pre-FEV_1_, %	56.7 (18–102)	55.5 (18–102)	62.5 (18–76)	0.79
Pre-FEV_1_/FVC	0.56 (0.31–0.72)	0.57 (0.31–0.72)	0.56 (0.35–0.7)	0.64
Post-FVC, L	2.7 (1.1–4.4)	2.7 (1.1–4.4)	2.7 (1.4–3.3)	0.29
Post-FVC, %	81 (42–130)	81.5 (42–130)	87 (43–96)	0.78
Post-FEV_1_, L	1.47 (0.53–2.82)	1.44 (0.67–2.82)	1.49 (0.53–2.09)	0.53
Post-FEV_1_, %	60 (21–105)	57 (22–105)	60 (21–79)	0.60
Post-FEV_1_/FVC	0.59 (0.35–0.73)	0.59 (0.35–0.73)	0.56 (0.37–0.70)	0.62
Pre-HR, beats/min	83 (57–109)	83.5 (57–109)	80.5 (60–98)	0.66
Post-HR, beats/min	107 (69–149)	108 (69–149)	105 (70–122)	0.48
Pre-Borg	0 (0–3)	0 (0–3)	1 (0–3)	0.06
Post-Borg	4 (1–7)	4 (2–7)	4 (1–7)	0.77
pre-spO_2_, %	95 (88–99)	95.5 (88–99)	95 (90–98)	0.31
post-spO_2_, %	90.5 (75–96)	90.5 (75–96)	90 (80–95)	0.61
pre-IC, ml	1.78 (0.92–2.77)	1.78 (1.07–2.77)	1.73 (0.92–2.04)	0.20
post-IC, ml	1.73 (0.94–2.83)	1.73 (0.97–2.83)	1.7 (0.94–2.43)	0.45
∆IC, ml	0 (− 1.1–0.55)	− 0.02 (− 0.49—0.55)	0.11(− 1.1–0.28)	0.37
∆spO2, %	− 4.5 (− 22 ~ 0)	− 4.5 (− 22 ~ 0)	− 4.0 (− 18 ~ 0)	0.97
Distance, m	456 (182–624)	468 (328–624)	408 (182–517)	0.03
DSP, m%	411.1 (149.2–536.6)	421.2 (255.6–536.6)	373.2 (149.2–464.6)	0.04

Data was presented as median (range)

Significantly difference between patients in the synchronized group and desynchronized group was defined as P < 0.05

*6MWT* six minutes walking test, *FVC* forced vital capacity, *FEV*_*1*_ forced expiratory volume in 1st second, *HR* heart rate, *∆IC* change of inspiratory capacity, *∆spO2* change of oxyhemoglobin saturation by pulse oximetry, *DSP* distance saturation product

**Table 3:** Main correlations with distance and distance saturation product (DSP) as assessed by 6MWT

Variable	Distance		DSP	
	r value	p value	r value	P value
Age, years	− 0.53	< 0.001	− 0.53	< 0.001
Synchrogram Index	0.42	0.001	0.41	0.001
BMI, kg/m^2^	− 0.07	0.62	− 0.05	0.69
Male, n (%)	0.13	0.34	0.17	0.21
CAT	− 0.44	< 0.001	− 0.44	< 0.001
mMRC	− 0.53	< 0.001	− 0.61	< 0.001
Smoking, n (%)	0.02	0.86	0.03	0.83
Presence of emphysema, n (%)	− 0.42	0.002	− 0.45	0.001
EF, %	− 0.12	0.39	− 0.13	0.35
E/e' ratio	− 0.34	0.06	− 0.35	0.06
Left atrial, mm	0.04	0.78	0.09	0.50
Presence of diastolic dysfunction	0.07	0.59	0.05	0.72
EOS count, number/l	0.08	0.59	0.10	0.45
IgE, pg/ml	− 0.13	0.37	− 0.11	0.43

Presence of emphysema was defined as presence of emphysema in the high-resolution computed tomography

Significant correlation was defined as P < 0.05

*6MWD* six minutes walking distance, *DSP* distance saturation product, *BMI* body mass index, *6MWT* six minutes walking test, *HS-CRP* high sensitivity C reactive protein, *EF* ejection fraction, *LA* left atrial, *CAT* chronic obstructive pulmonary disease assessment test, *mMRC* modified medical research council, *E/e′ ratio* the ratio of the trans-mitral early peak velocity over early diastolic mitral annulus velocity, *EOS.count* eosinophil count

**Table 4:** Multivariable regression model for distance as assessed by 6MWT

variable	Beta	SE	t value	p-value
Age, year	− 3.1	1.2	− 2.6	0.01
Synchrogram Index	93.3	42.2	2.2	0.03
CAT score	− 1.4	1.7	− 0.8	0.41
mMRC	− 37.9	12.9	− 2.9	0.005
Presence of emphysema	− 38.6	21.2	− 1.8	0.08

Presence of emphysema was defined as presence of emphysema in the high-resolution computed tomography

r^2^ = 0.56, adjusted r^2^ = 0.51, Residual stand error = 60.9, p < 0.0001

*SE* stand error of beta

The fitted model is Distance = 671.3 + 93.3(Synchrogram Index) − 3.1Age − 37.9mMRC

**Table 5:** Multivariable regression model for distance saturation product (DSP) as assessed by 6MWT

variable	Beta	SE	t value	p-value
Age, year	− 2.8	1.1	− 2.4	0.02
Synchrogram Index	89.1	39.0	2.3	0.03
CAT score	− 0.9	1.6	− 0.6	0.57
mMRC	− 40.7	14.3	− 2.8	0.007
Presence of emphysema	− 41.1	19.6	− 2.1	0.04

Presence of emphysema was defined as presence of emphysema in the high-resolution computed tomography

r^2^ = 0.63, adjusted r^2^ = 0.58, residual stand error = 56.3, p < 0.0001

*DSP* Distance saturation product, *SE* stand error of beta

The fitted model is DSP = 619.2 + 89.1(Synchrogram Index) − 2.8Age − 50.7mMRC − 41.1Emphysema

## Discussion

This is the first study to study CRC of patients with COPD by clustering them into synchronized or desynchronized groups. Patients in the synchronized group had higher 6MWD and DSP compared with those in the desynchronized group. Our results identified the synchrogram index as a novel independent variable by which to predict DSP and 6MWD, which is a well-established predictor of mortality [38] and acute exacerbation [39] in patients with COPD. The synchrogram index depicts the heart–lung interaction; therefore, its relationship with the 6MWD suggests that it could potentially provide clinically useful information from a dimension other than 6MWD. Confirming the clinical applicability of this index to COPD patients (e.g., predicting mortality or acute exacerbation) will require following up patients for an extended period of time. The fact that CRC can be easily obtained using widely available non-invasive equipment means that it is applicable to a variety of healthcare environments, such as long-term homecare monitoring with the assistance of mobile technologies.

Researchers have previously demonstrated that 6MWD is an important predictor of survival in COPD patients [40, 41]. The limited walking distance demonstrated by COPD patients can be attributed to age [42], impaired heart function with low ventricular ejection fraction (LVEF < 50%) [43], and impaired respiratory function including desaturation [44], emphysema severity [45], dyspnea scores [46], inspiratory capacity, and dynamic hyperinflation [47]. Several comorbid conditions, such as skeletal muscle dysfunction, impaired autonomic regulation, and nutritional factors, also contribute to exercise intolerance in patients with COPD [48, 49].

Our study identified a correlation between 6MWD and age, mMRC, and synchrogram index. Since the severity of emphysema, desaturation, and diastolic heart failure do not show a significant contribution in our patients, we suggest that impaired pulmonary and heart function are not directly related to 6MWD in this study. Rather, we should consider nutritional status [50], oxygen utilization by peripheral muscles, and/or negative cardiorespiratory-muscle interactions [51]. Previous studies posited that integrated cardiopulmonary function and muscle condition could reflect 6MWD [49]. These assertions are in line with our findings indicating that the synchrogram index (a quantification of heart–lung interactions), is an independent factor contributing to 6MWD. These findings warrant further investigation into the relationships among oxygen utilization by peripheral muscle, cardiopulmonary-muscle interactions, and muscle strength.

DSP is a reliable factor to predict mortality among patients with bronchiectasis [52], interstitial lung disease [53], and COPD [54, 55]. In this study, patients in the desynchronized group present a lower DSP, implying an elevated likelihood of poor outcomes but need adequate follow-up duration to confirm. To our knowledge, this is the first study to evaluate factors that associated with DSP in COPD patients. Age, mMRC, synchrogam index and emphysema are independent variables to predict DSP. Emphysema is an independent factor in determining DSP but not 6MWD in this study, which may be related to the correlation of emphysema among desaturation during exercise [56] and its contribution to the desaturation component of DSP.

A strong heart–lung interaction may improve ventilation and perfusion matching, resulting in a better oxygen transport [57]. However, we did not observe any discrepancy between the synchronized and desynchronized groups in terms of saturation. This may be explained by the fact that we excluded patients who were using oxygen daily and by the reason that there was similar proportion of emphysema. Note that there may be a link between desaturation and coupling in those patients. In order to evaluate this relationship, it is necessary to explore COPD patients with chronic hypoxemic failure in the next program.

This study faced a few limitations. First, despite measuring and quantifying the coupling between respiration flow signals and IHR, we cannot conclude causality. Second, strict inclusion criteria prevented us from analyzing patients who were using oxygen on a daily basis, with the result that the study population was small, particularly in the desynchronized subgroup. Third, most of the patients in this study were male, with low CAT scores, and of East Asian descent, such that our findings are not necessarily generalizable to all COPD patients. Finally, this is a cross-sectional and preliminary data of a prospective-designed study. Due to the insufficient follow-up time, we cannot evaluate mortality outcomes and cardiac vascular events. We will continue monitoring the subjects in this study in order to observe the clinical impact of synchronization in heart–lung interactions.

## Conclusions

This study first conducted the CRC analysis to describe heart–lung interactions of COPD patients. Asides from age and mMRC, synchrogram index is an independent variable that could predict 6MWD and DSP.

## Data Availability

The data sets analyzed during the current study are available from the corresponding author upon reasonable request.

## Abbreviations

ACO: Asthma-COPD overlap
AE: Acute exacerbation
BMI: Body mass index
CAT: Chronic obstructive pulmonary disease assessment test
COPD: Chronic obstructive pulmonary disease
CRC: Cardiorespiratory coupling
ECG: Electrocardiogram
ETCO_2_ sensor: End tidal CO_2_ sensor
E/e’ ratio: The ratio of the transmitral early peak velocity over early diastolic mitral annulus velocity
FEV_1_: Forced expiratory volume in 1st second
FVC: Forced vital capacity
GOLD: Global Initiative for Obstructive Lung Disease Criteria
HFmrEF: Heart failure with mid-range ejection fraction
HFlowEF: Heart failure with low ejection fraction
HRV: Heart rate variability
HS-CRP: High sensitivity C-reactive protein
ICS: Inhaled corticosteroids
IHR: Instantaneous heart rate
mMRC: Modified medical research council
LABA: Long-acting beta agonists
LAMA: Long-acting antimuscarinic agents
OCS: Oral corticosteroids
SST: Synchrosqueezing transform
